# Are modern voice prostheses better? A lifetime comparison of 749 voice prostheses

**DOI:** 10.1007/s00405-013-2611-0

**Published:** 2013-06-29

**Authors:** P. Kress, P. Schäfer, F. P. Schwerdtfeger, S. Rösler

**Affiliations:** 1 Klinikum Mutterhaus der Borromaerinnen, Feldstrasse 16, 54290 Trier, Germany; 2 University of Applied Science Esslingen, Esslingen, Germany

**Keywords:** Laryngectomy, Device life time, Voice prosthesis, Provox Vega, Blom-Singer

## Abstract

The aim of the study was to compare device life of more recent indwelling voice prostheses Provox Vega and Blom-Singer Dual Valve to device life of well-known standard devices (Provox 2, Blom-Singer Classic). In a prospective, non-randomised study, device life of Blom-Singer Classic, Blom-Singer Dual Valve, Provox2, Provox Vega and Provox ActiValve voice prostheses was recorded in a group of 102 laryngectomised patients. In total 749 voice prosthesis were included. Average overall life time was 108 days, median 74 days. The prosthesis with the longest dwell time was the Provox ActiValve (median 291 days). Provox Vega had longer device life compared with Provox2 (median 92 days vs 66 days; *p* = 0.006) and compared with Blom-Singer Classic (median 92 days vs 69 days; *p* = 0.004). In conclusion, device lifetimes of Provox Vega and ActiValve were better than those of Provox2 and the Blom-Singer Classic. New voice prostheses, with a defined valve opening pressure (Provox Vega, Provox ActiValve, Blom-Singer Dual Valve) had longer lifetimes than prostheses without a defined opening pressure (Blom-Singer Classic and Provox 2).

## Introduction

In 1982, the first indwelling voice prosthesis, the “Groningen Button” was introduced [1]. From then on, the indwelling concept of voice restoration became the gold standard of tracheoesophageal voice restoration in industrial nations as it allows supplying also older and less skilful patients with voice prostheses [1]. Disadvantages of the indwelling concept are the need for a physician or a speech language pathologist (SLP) to change the voice prostheses and the need for a placement tool. Moreover, the device life of indwelling voice prostheses is largely dependent on patients and prosthesis factors (e.g. speaking habits, diet, biofilm resistance, underpressure, valve features) and cannot be improved by cleaning strategies as they are not cleaned on a daily basis similar to non-indwelling devices. Placement tools became more and more sophisticated over the years, evolving from retrograde placement (Provox 1 1990, Groningen ULR) [2] to anterograde placement (Blom-Singer Classic with a Gel Cap, Provox 2 with an inserter) [3, 4] and most recently to exactly controlled anterograde placement with the third-generation Provox Vega with the SmartInserter [5] and the Provox Vega Puncture Set (for all-in-one surgical creation of the TE puncture and placement of the voice prosthesis) [6]. In Germany indwelling voice prostheses with an anterograde insertion method and a rational cost/lifetime ratio became standard devices (Provox 2, Blom-Singer Classic). Due to patient comfort, safety and economical aspects, in the past years, several voice prostheses with additional features intended to prolong device life have become available. The Blom-Singer Advantage with a valve flap containing 7 % of silveroxide for patients with premature device failure due to biofilm formation colonisation [7, 8] and the Blom-Singer Dual Valve that incorporates two valve flaps containing silveroxide in the shaft of the device (no clinical data are currently available for this device), and the Provox ActiValve in which the valve flap and valve seat are constructed out of fluoroplastic and valve flap closure is supported by magnets for patients with early device failure due to biofilm formation and/or underpressure in the oesophagus during swallowing and/or inhalation [9]. In addition to these design changes, newer voice prostheses (Blom-Singer Dual Valve, Provox Vega and ActiValve) have also been found to have more defined opening pressures, which may lead to differences in device life [10].

Besides ease of phonation, overall voice quality and patient preference, device lifetime is an important parameter measured in several studies comparing different voice prostheses. It is generally considered an important factor from a cost perspective; the shorter the device life, the higher the consumption of prostheses and hospital visits.

Studies to date have revealed that, on average, the device life of a standard indwelling voice prosthesis falls somewhere between 4 and 6 months for the majority of patients [2, 11–14]. However, significant variations in device life have been reported within patients, between different patient groups, and across device types, influenced by reflux, nutrition and geographical regions [15, 16]. Our study can rule out effects of different patient groups and allows comparison of device life time without the influence of socioeconomic and reimbursement aspects [15, 16]. Device life of the Provox2 has been studied in a variety of studies and often serves as a reference for comparison with other studies and other types of voice prostheses [2, 12–14]. The device life of the Provox ActiValve has been shown to be substantially longer than that of the traditional indwelling voice prostheses [9, 17, 18]. Also the Blom-Singer Advantage (model with hard valve assembly) has been shown to have a longer device life in selected patients [7, 8]. Published results regarding the device life of the Provox Vega have mainly covered short observation periods and were found to be similar to the device life of Provox2 [15, 16]. However, one long-term study from Australia indicates that the Provox Vega may have a rather long device life, at least in the Australian setting [19]. To our knowledge no device life data have been published yet regarding to the Blom-Singer Dual Valve. To date, very few studies have investigated differences in device life between the various indwelling devices, and the outcomes have not shown large differences, except for the Provox ActiValve, compared to the standard indwelling devices [20–23].

The most common reason for replacement of an indwelling device is leakage through the device. Other reasons for replacement are for example the need for size changes, increased speaking effort, granulation tissue and inflammation/infection (‘device-related’ reasons) [12, 22]. Reasons for diversity in device life duration such as patient- and treatment characteristics as well as socioeconomic and reimbursement aspects are also focus of research [19].

In our centre a variety of voice prostheses from different manufacturers is being used to meet each patient’s individual needs. Since device life is an important factor in clinical and economical decision making, the aim of our prospective study was to investigate and compare the device life of five different indwelling devices used at our institute with a special focus to compare the newer devices (Provox Vega and Blom-Singer Dual Valve) to standard prostheses used in our institution.

## Subjects and methods

### Subjects

All laryngectomised patients visiting the outpatient clinic of our hospital between November 2009 and November 2012 for a voice prosthesis change were entered in the study if they consented to the study and data privacy protocol. A total of 749 voice prosthesis replacements were included from a group of 102 laryngectomised patients. Ages ranged from 42 to 86 years, with a median of 64.4 years in female and 61.2 years in male patients (See Table 1).

**Table 1:** Patient characteristics

Characteristics	*N* = 102
Gender	
Male	91
Female	11
Age	
Range	42–86 years
Median male	61.2
Median female	64.4
Tumour localisation	
Larynx	58
Hypopharynx	29
Oropharynx	8
Cervical oesophagus	3
Others/unclear	4
Type of surgery	
Laryngectomy	75
Pharyngolaryngectomy	18
Unknown	9
Reason total laryngectomy	
Initial treatment	65
Salvage laryngectomy	33
Unclear	4
Radiotherapy	
Primary treatment	33
Post-operative	58
None	7
Unknown	4
Postoperative follow-up	
Range	3 months–23 years
Median	6.8 years

Patients were seen by one of the 17 physicians tending the outpatients of one centre in Trier, Germany. The physicians had different levels of experience, and five different voice prostheses (Provox2, Provox Vega 22.5 Fr, Provox ActiValve, Blom-Singer Classic Indwelling 20 Fr, Blom-Singer Dual Valve 20 Fr) were used. Excluded were cases where the device failed due to “patient/puncture related reasons” (e.g. puncture infection, size changes and dislocation of the prosthesis). Also excluded were the first prostheses used intraoperatively, since it is known that their service life can be disproportionately long [2, 24]. Included were cases where the device failed due to “prostheses related reasons” (e.g. leakage through the device, high pressure speech, and biofilm growth on the outside of the shaft). Leakage of the valve was assumed when reported by the patient or when it was detected during a swallowing test (three swallows of clear water over a period of 2 min).

All voice prostheses were used in accordance with the manufacturer’s recommended use of the device and the manufacturer’s recommended insertion method. The following algorithm of prosthesis choice is generally accepted and applied in our institution: Provox 2, Provox Vega and Blom-Singer Classic are used as standard devices by choice of the doctor using the device based on his experience and patients requirements (e.g. stoma size, place of TE puncture, preferred insertion method, need for overshooting, speaking problems). If a standard device shows a reduced device life <6 weeks more than two times, a moderately expensive special prosthesis is used (Blom-Singer Dual Valve, hard valve assembly). Does device life improve to 3 months or more without negative side effects (e.g. speaking problems), the patient is consecutively fitted with this type of voice prosthesis. If lifetime does not improve to at least 2 months, the patient is considered to be fitted with the effective but also expensive Provox ActiValve. At the time the voice prosthesis had to be removed, the life of the voice prosthesis was calculated in days, the type of prosthesis was listed and the history was checked to exclude that in the meantime no undocumented voice prosthesis change (e.g. during emergency services) had taken place.

### Short description of each device

#### Blom-Singer^®^ Classic (20 French (Fr) and 16 French), Inhealth Technologies, Carpinteria, CA, USA

**Fig. 1:**
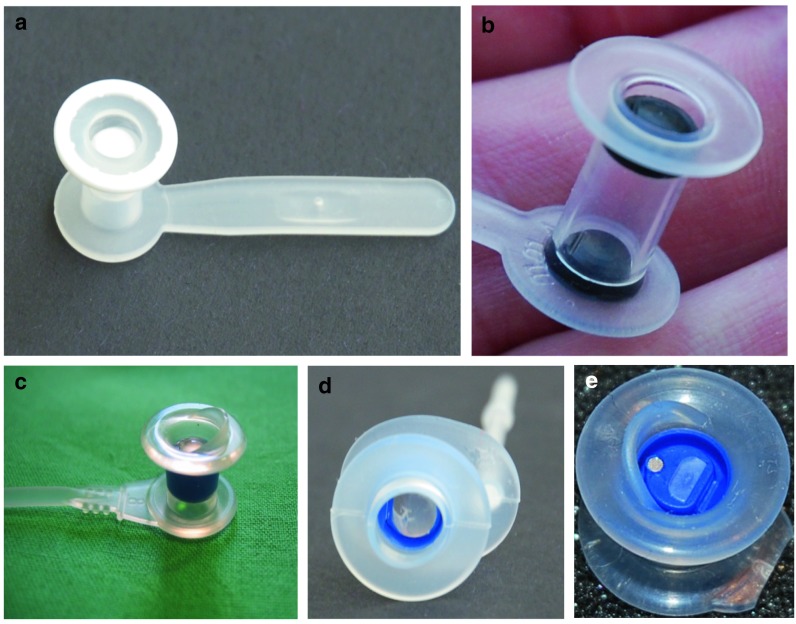
Voice prostheses (Photos courtesy of Dr. P Kress) **a** Blom-Singer^®^ Classic **b** Blom-Singer^®^ Dual Valve **c** Provox^®^2 **d** Provox^®^ Vega **e** Provox^®^ ActiValve™

This soft and flexible voice prosthesis is entirely made out of silicone, with a flap valve incorporated in the shaft. The outer ring of the oesophageal flange is radiopaque. The shaft diameter used in our clinic is 20 Fr. It is inserted anterograde, with a gel cap. It is used in our clinic as a standard prosthesis and for the management of complications as it is available in oversized shaft lengths (up to 30 mm). See Fig. 1a.

#### Blom-Singer^®^ Dual Valve™, Inhealth Technologies, Carpinteria, CA, USA

The Blom-Singer Dual Valve has a silicone housing and two silicone flap valves containing 7 % of silveroxide mixed into the silicone, one on the oesophageal side, and an additional one on the tracheal side. The double valve is intended to increase the lifetime, because it is assumed that the second valve will prevent leakage after the first valve fails. In addition, the silveroxide is expected to have antifungal properties. Speaking pressures and valve opening pressures of the Blom-Singer Dual Valve are reported to be higher than in the Blom-Singer Classic 20 Fr. Blom-Singer Dual Valve prostheses were used with 20 Fr shaft diameter and inserted with the gel cap method. See Fig. 1b.

#### Provox^®^ 2, Atos medical, Hörby, Sweden

The Provox2 voice prosthesis is made out of medical grade silicone, with a radiopaque valve seat made out of fluoroplastic, and a silicone flap valve. The device is inserted anterograde with an inserter pin and loading tube. The outer diameter is 22.5 Fr. This prosthesis came on the market in 1997 and has been used in our clinic as a standard prosthesis since it was first introduced. See Fig. 1c.

#### Provox^®^ Vega™ 22.5, Atos Medical, Hörby, Sweden

The Provox Vega prosthesis is the technically improved successor of the Provox2 prosthesis. It is specifically designed to have good airflow characteristics and precise valve characteristics. The tracheal flange is oval, designed to better fit the tracheal anatomy and prevent prosthesis rotation. The safety strap is attached originating in a 90° angle from the tracheal flange as in the Provox1 to eliminate the risk of tracheal mucosa injuries and granulation. It is available in three outer diameters (17 Fr, 20 Fr and 22.5 Fr). In the present study the 22.5 Fr was used because it has the best aerodynamic properties. The valve of the Provox Vega is designed so that it opens in a defined opening pressure range and unintended valve openings (e.g. during inspiration) can be reduced [10]. The Provox Vega comes preloaded in a new insertion system (SmartInserter™). The SmartInserter prevents unintentional overshooting (placing the entire prosthesis in the oesophagus) which saves physician and patient unpleasant flange repositioning procedures. See Fig. 1d.

#### Provox^®^ ActiValve™, Atos Medical, Hörby, Sweden

The Provox ActiValve voice prosthesis was developed with the aim of solving problems in a select patient group that is experiencing extremely short device lifetimes due to excessive biofilm growth or underpressure in the oesophagus during swallowing or inhalation. The prosthesis has a housing of medical grade silicone, similar to the Provox2. Both the valve seat and the valve flap are made out of fluoroplastic, using magnets available in three different strengths to support valve closure. Outer diameter and available lengths are equal to Provox2. Due to significantly higher costs of the ActiValve, it is only used in our clinic for the management of extremely short device life. In the current study, we have provided patients a Provox ActiValve when their voice prosthesis three times in a row had a life of 40 days or less, the shaft length of the prosthesis was always the same and there was no trachea-oesophageal (TE) puncture pathology. In general, the magnetic strength “strong” was selected. If the patient experienced speech difficulties, we changed to the strength version “light”. If the patient was still experiencing air filling of the stomach or a short device life time, we changed to “extra strong”. Provox ActiValve users in our clinic are advised that if the device is still in situ after 1 year, it should be replaced regardless of whether it is leaking or not. See Fig. 1e.

### Statistical analysis

Non-parametric tests (Mann–Whitney) were used to compare the median device lifetimes in days. Median lifetimes are more informative compared to means, as suggested by Op de Coul et al. [12]. Boxplots and Kaplan–Meier survival curves were created to show the lifetimes. A logrank test (Mantel–Cox) was used to compare the devices overall, truncated at 1 year, 6 and 3 months. For all analyses, we used SPSS version 19.0 and significance was set at *p* < 0.05.

## Results

**Fig. 2:**
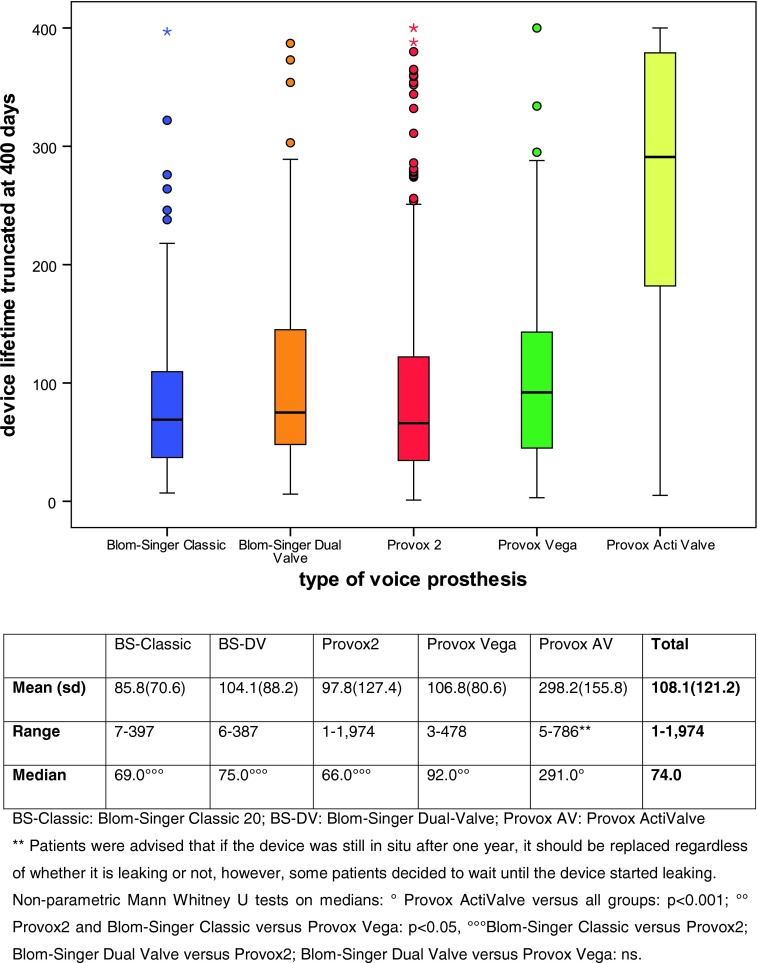
Boxplots with device lifetimes, all prostheses (this graph has been cut off at 400 days to enhance visibility)

In total, 749 voice prostheses were included, used by 102 patients; 108 Blom-Singer Classic Indwelling, 62 Blom-Singer Dual Valve, 424 Provox2, 117 Provox Vega, and 38 Provox ActiValve. Per device, the mean and median were, respectively: Blom-Singer Classic 86/69 days, Blom-Singer Dual Valve 104/75 days, Provox2 98/66 days, Provox Vega 107/92 days and Provox ActiValve 298/291 days (See Fig. 2).

Provox2 was the most frequently used voice prosthesis (62 %), because this prosthesis was the only Provox standard prosthesis in the beginning of this study. During the study period the Provox2 got more and more replaced by the Provox Vega. The prosthesis with the longest dwell time was the Provox ActiValve; this device appeared to have at least three times longer lifetimes compared to the other devices, and its device life time was significantly longer than any of the other standard voice prostheses (*P* < 0.0001).

When comparing the medians within the groups, Provox ActiValve had significantly longer lifetimes compared to all other prostheses (*p* < 0.001). In the group of standard voice prostheses the Provox Vega had significant longer lifetimes compared to Provox2 (*p* = 0.006) and compared to Blom-Singer Classic (*p* = 0.004). There was no significant difference between the device life of Blom-Singer Classic versus Provox2 (*p* = 0.604), Blom-Singer Dual Valve versus Provox2 (*p* = 0.233) and versus Provox Vega (*p* = 0.159). (See table accompanying Fig. 2).

Prostheses with a defined valve opening pressure (Blom-Singer Dual Valve, Provox Vega and ActiValve) had longer lifetimes than prostheses without a defined opening pressure (Blom-Singer Classic and Provox 2).

**Fig. 3:**
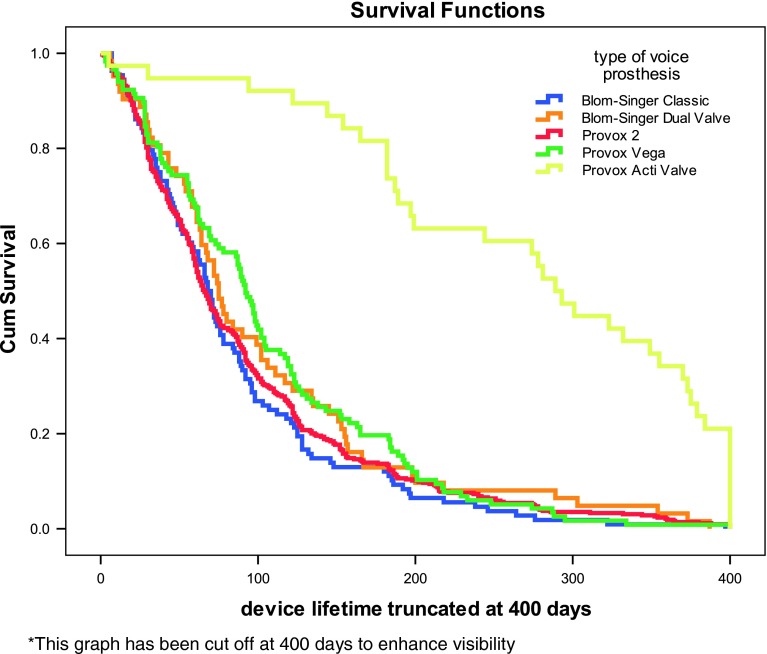
Kaplan–Meier curves, all prostheses (this graph has been cut off at 400 days to enhance visibility)

Figure 3 shows the Kaplan–Meier curve of the lifetimes per device, logrank *p* < 0.001. When removing Provox ActiValve out the analysis, the logrank truncated at 1 year, 6 and 3 months was, respectively, *p* = 0.181, *p* = 0.088 and *p* = 0.024. Provox Vega appeared to have significant longer lifetimes compared to Provox2, truncated at 1 year (*p* = 0.133), 6 months (*p* = 0.024) and 3 months (*p* = 0.005), and compared to Blom-Singer Classic, truncated at 1 year (*p* = 0.043), 6 months (*p* = 0.022), and at 3 months (*p* = 0.006) (Fig. 3).

## Discussion

Among 102 patients, in total 749 voice prostheses were included. The average life time for all devices was 108 days, median 74 days (logrank *p* < 0.001). The prosthesis with the longest dwell time was the Provox ActiValve. Most interesting for the clinical use, the Provox Vega showed to have a significantly longer lifetime compared to Provox2 and to Blom-Singer Classic. Therefore, we recommend changing from Provox 2 to Provox Vega whenever possible. Prostheses with a defined valve opening pressure (Blom-Singer Dual Valve, Provox Vega and ActiValve) had longer lifetimes than prostheses without a defined opening pressure (Blom-Singer Classic and Provox 2). This underlines the importance of aiming to prevent unintended valve flap openings during inspiration by using a voice prosthesis with a defined valve opening pressure if a long device life is intended.

Compared to other studies [2, 12–14], the current study shows an overall relatively short device lifetime for the ‘standard’ (Provox2, Provox Vega, Blom-Singer Classic) devices used in our patient population (on average 3.2 months instead of 4–6 months). This is most likely due to the fact that prostheses are removed in our clinic at the very first signs of valve failure and patients are trained not to tolerate any leakage. Moreover, an overrepresentation of devices with a short lifetime during the observation period may be present. The data for this study were collected November 2009 and November 2012 and all replacements during this time frame are included in the analyses. Therefore patients with short device life have contributed several devices to the sample whereas patients with a long device life only 1 or 2 devices. We do however believe that our data display clinical reality more precisely than others, as they are based on a medically safe definition of leakage and economic effects can more or less be ruled out (patients do not pay for their prostheses on an individual basis).

The long device life of the Provox ActiValve in comparison with the ‘standard’ devices is in concordance with other studies [9, 17, 18]. This is not surprising as this device is considered a ‘problem-solver’, specifically developed for laryngectomised patients with early device failure. This device is priced higher than the standard voice prostheses. Despite the higher costs, the use of this device could be cost-effective not to mention the positive effect on patient safety and comfort. The actual observed lifetime of the ActiValve may be even longer than the average of 298 days or median of 291 days reported in our study (compared to median 337 days reported by Soolsma et al. and a mean of 300 days reported by Graville et al. [17, 18]), as in our clinic this device is usually changed prophylactically if it is still in situ at 1 year to prevent biofilm colonisation of the TE puncture and not because of valve defects. Our results show that in comparison with the other devices used, the lifetime of the Provox ActiValve was about three times longer. Keeping in mind that this device is in our clinic used in patients with a very short device life (i.e. about 1 month), it can be expected that—as in the literature [9]—also in our setting a 14–16-fold increase in lifetime is found when compared within the same patient.

This is the first study to report on device life of the Blom-Singer Dual Valve. In our clinic, the Blom-Singer Dual Valve was used to address early device failure. Our results did not reveal a significant difference between the device life of the Blom-Singer Dual Valve (median 75 days) and the Blom-Singer Classic (median 69 days, *p* = 0.202), or the Provox2 (median 66 days, *p* = 0.233), or the Provox Vega (median 92 days, *p* = 0.159). But similar to the Provox ActiValve, this device was used in patients with short device life (>2 subsequent short device life) and as such, if the average were compared within the same patient, an improvement in device life may be found. Comparison with results for the Provox ActiValve (median 291 days, *p* < 0.001), shows a markedly shorter device life which is in concordance with its lower valve opening pressure and price. The device life of the Blom-Singer Dual Valve in our study also seems to be somewhat lower than the device life reported in previous investigations with the Blom-Singer^*®*^ Advantage (Kress: mean 101, median 87 days, Leder: mean 118–168 days) [7, 8].

The average device life for Provox2 devices was compared to the literature quite low (mean 88 days versus 111–163 days in four comparable studies) [12, 13, 25, 26]. However, another German retrospective study conducted from 1993 to 1999 analysed the device life for amongst others Provox2, where the 96 days on average was nearly equal compared to our observation [24]. These findings could be explained by the above mentioned strict definition of leakage, the easy available health care provider in Germany, and the almost non-existing economic impact on the patient that asks for a new prosthesis.

The Provox Vega having longer life times compared to Provox2 is a new finding in the current study compared to some of the previous literature [15, 16, 23]. These previous studies covered short observation periods, whereas the current study covers a long observation period, allowing more time to observe longer dwell times of the different devices. This is confirmed by a long-term (2 years) device life study on Provox Vega devices in an Australian setting, which reported a median of 222 days and an average of 207 days. These dwell times are considerably longer than those reported in our study, (median of 92 days and average of 107 days), which might be explained by health economical and geographical differences [19].

The device life of the Blom-Singer Classic found in our study was also quite low compared to others. A mean 68 versus 107 days in a study reported by Schafer et al. and 143.5 days reported by Trussart et al. [20, 24]. This could be due to the fact that Blom-Singer Classic prostheses are frequently used for the management of puncture complications and might in some cases have not been correctly eliminated from the data pool.

A limitation of the current study is that it was not possible to compare lifetimes for the different devices within the same patient. In our setup, one patient could have used several different devices, or only one type of device, based on the choice of the physician at the time of replacement. Another point of attention might be the relatively limited number of the ActiValve devices used in the current study (*n* = 38, 5.5 % of total) that is based on the high price and strict criteria for its use.

In conclusion, our study can rule out effects of different patient groups and allows comparison of device life time with very limited influence of socioeconomic and reimbursement aspects. It shows that the device lifetime of Provox Vega was better than that of Provox2 and that of Blom-Singer Classic devices. For further developments on voice prostheses it should be considered, that devices with a defined valve opening pressure (Blom-Singer Dual Valve, Provox Vega and ActiValve) had longer lifetimes than prostheses with a low and undefined opening pressure (Blom-Singer Classic and Provox 2).
